# Evaluation of 10-Year Experience of Wegener's Granulomatosis in Iranian Children

**DOI:** 10.1155/2013/694928

**Published:** 2013-07-15

**Authors:** Fatemeh Tahghighi, Mohamad-Hassan Moradinejad, Yahya Aghighi, Reza Shiari, Seyed-Reza Raeeskarami, Farhad Salehzadeh, Vadood Javadi, Vahid Ziaee

**Affiliations:** ^1^Pediatric Rheumatology Research Group, Rheumatology Research Center, Tehran University of Medical Sciences, Tehran, Iran; ^2^Growth and Development Research Center, Tehran University of Medical Sciences, Tehran, Iran; ^3^Department of Pediatrics, Tehran University of Medical Sciences, Tehran, Iran; ^4^Department of Pediatrics, Shahid Beheshti University of Medical Sciences, Tehran, Iran; ^5^Department of Pediatrics, Ardabil University of Medical Sciences, Ardabil, Iran; ^6^Division of Pediatric Rheumatology, Children's Medical Center, No. 62 Dr. Gharib Sreet, Keshavarz Boulevard, Tehran 14194, Iran

## Abstract

Wegener's granulomatosis or granulomatosis polyangiitis (GPA) is an uncommon chronic systemic vasculitis in children. The aim of this study was to describe pediatric patients with GPA in Iran. We studied records of all patients with GPA diagnosis who were referred to all Iranian Pediatric Rheumatology Division from 2002 to 2011. A total of 11 patients (5 females and 6 males) enrolled in this study. In children less than 15 years old, the prevalence of GPA is 0.6 per million. The mean age of GPA diagnosis was 11 years and average delay diagnosis was 20 months. Mortality rate was 18.1% due to pulmonary vasculitis and infection. The most common organ system involvement was upper and lower respiratory tract involvement (81.8% and 63.9%, resp.). Other common manifestations were renal (36.3), skin (27.2%), and eye involvement (18.2%).

## 1. Introduction

Wegener's granulomatosis (WG) is a chronic systemic vasculitis mainly affecting the upper and lower respiratory tracts and kidneys that usually involve small to medium-sized arteries [1]. This condition that also known as granulomatosis polyangiitis (GPA) was described by McBride in 1897 as a midfacial granuloma syndrome, but the complete picture was described in 1930s [2, 3]. This vasculitis was characterized by the formation of necrotising granuloma in the respiratory tracts and necrotizing pauci-immune glomerulonephritis. The triad of upper and lower respiratory tract inflammation and renal disease are characteristic for GPA. 

The etiology is unknown, but it is usually associated with antineutrophil cytoplasmic antibody (ANCA) that was detected in predominantly cytoplasmic form (cANCA). The peak incidence of GPA is in the fourth to sixth decades [4–6] and it is rare in children, so both the American college of Rheumatology (ACR) classification criteria [7] and the subsequent Chapel Hill consensus conference (CHCC) disease definitions [8] are based largely on adult data. Recently, a consensus committee of pediatric rheumatology and nephrology experts, under the auspices of the European League Against Rheumatism (EULAR) and the Pediatric Rheumatology European Society (PReS), proposed a system of classification for vasculitis that took into account existing pediatric knowledge and experience [9]. 

At onset, nonspecific complaints of fever, malaise, fatigue, and weight loss are very common. A large majority of children with GPA present with multiple organ involvement. 

There is no comprehensive study on GPA in Iran. So, the aim of this study was to describe pediatric patients with GPA from all pediatrics rheumatology divisions of Iran to analyse the variety of clinical manifestations seen and to compare the data with other published series.

## 2. Material and Methods

We studied records of all patients with GPA diagnosis who were referred to all Iranian Pediatric Rheumatology Division from 2002 to 2011. Data included age, gender, age of onset, delay in diagnosis, clinical features before diagnosis, and clinical signs observed at diagnosis, laboratory and histopathological findings, imaging findings, and response to treatment. We used the EULAR/PRES criteria [9] to confirm the diagnosis. The patient was considered to have GPA when three of the following six criteria were present:

1. nasal, oral inflammation, or sinus inflammation;
2. abnormal chest radiograph or chest CT scan;
3. abnormal urinalysis including signification proteinuria;
4. granulomatous inflammation on biopsy or necrotizing pauci-immune GN on biopsy;
5. subglottic, tracheal, or endobronchial stenosis
6. PR3 ANCA or cANCA staining.

This study was approved by local ethical committee of Children's Medical Center, pediatric center of excellence in Iran. 

## 3. Result

During 10 years of study, 11 patients with GPA diagnosis were admitted in all Iranian Pediatric Rheumatology Division. Five patients were female (45.4) and 6 male (54.5) with male/female ratio of 1.2 : 1. During this period, almost one third of the population (17,680,000) is less than 15 years old. The prevalence of GPA was 0.6 per million. The mean age at primary symptom onset was 9.7 (range 4 to 15) years and the mean age of GPA diagnosis was 11 (range 6 to 15) years. The average delay diagnosis was 20 months; this index was 6 months and 23 month in died and alive patients, respectively. Ten of 11 children fulfilled EULAR/PRES criteria and one patient was diagnosed based on the clinical picture typical of the disease. The mean duration of follow-up for living patients was 6.4 years. Two patients died (mortality rate 18.1%) including a female patient after 2 weeks of diagnosis with pulmonary hemorrhage and another male patient after 12 months of diagnosis with proptosis and orbital pseudotumour. Final diagnosis for the last patient was disseminated fungal infection after retroorbital granulomatous mass due to fungal infection. 

Initial clinical manifestations of our patients are summarized in Table 1. 

At onset, fatigue as a nonspecific complaint was very common (81.8%). The most common organ system involvement was upper respiratory tract (81.8%) and lower respiratory tract involvement was seen in 63.9%. Six children (54.4%) had abnormal imaging in chest X-ray or chest CT-scan. Renal involvement was observed in 4 children (36.3). 

The eyes involvement was seen in 2 patients, one of them had proptosis due to a retroorbital granulomatous mass (showed on biopsy), and the other patient had periorbital cellulitis.

Skin involvement was observed in 3 children. Two patients had necrotic lesions that one of them presented with pyoderma gangrenosum. In skin biopsy, they showed necrotizing granuloma and leukocytoclastic vasculitis and 3rd one had a nonspecific rash. Gastrointestinal involvement was found in 3 patients that one of them showed follicular gastritis in biopsy.

*Laboratory Markers*. Cytoplasmic antineutrophil cytoplasmic autoantibodies (cANCA) or proteinase-3 (PR3)-ANCA was positive in all patients and p-ANCA (MPO) was positive in 2 patients. ANA was positive just in one patient. Two patients had thrombocytopenia (67000 and 95000) without active bleeding. Antiplatelet antibody was negative in thrombocytopenic patients.

## 4. Discussion

The aim of this study was a comprehensive report on GPA in Iranian children. The peak incidence of GPA is in the fourth to sixth decades [4–6] and it is rare in children and the main reports on WG are in adult patients. Mortality rate in our study was 18% and it occurred during 1 year after diagnosis. This rate was 20.8% and 27% in other studies [10, 11]. Luqmani et al. reported higher mortality rate during the first year after diagnosis [10]. Similar to Luqmani et al study, the causes of mortality in our study were infection and vasculitis, but unlike their findings anyone of our patients didn't have renal failure [10]. Some factors have been studied as predictors of mortality in GPA. Higher age (>52 y) and renal involvement have been a positive effect on mortality rate, while the ENT involvement has had an association with a longer survival [10, 11]. 

Onset of GPA before 10 years old is uncommon. The mean age at diagnosis in our group was 11 years (rang 4 to 15) that was older than reported by Belostotsky et al. (6 years) [12]. The mean age at GPA diagnosis was reported 14.2 years (range 4 to 17) by Cabral et al. [13]. 

In our study, male/female ratio was similar to that reported by Stegmayr et al. [14] and adult literature [15–17], but some studies reported a significant female predominance in their patients [12, 18–21]. 

Similar to other studies, fatigue and malaise were the most common symptom in our patients [21, 22]. Respiratory tract is the most common involvement in GPA. In our studies, upper and lower respiratory tract was affected in 81.1% and 63.3% patients, respectively. This rate was very different in other studies. Chinese patients had 51.9% and 77.8% [23] and Rodrigues et al. [20] reported 64% and 36% upper and lower respiratory tract involvement, respectively. Sometimes respiratory tract involvement needs surgical intervention. Eustaquio et al. reported 25% respiratory tract involvement with airway lesions and surgical interventions [17].

In Belostotsky study renal involvement was 3 times in children after 5 years in comparison to less than 5 years old [12]. Renal involvement has been reported very different in children between 53 and 88% [12, 23] and this rate was less frequent than in adult (85%) [15]. It seems that renal involvement is more frequent in adult than in children. In addition, early diagnosis and treatment may prevent nephritis and progress to end stage renal disease. Similar to Cabral report, in our group microscopic hematuria was the most common involvement [13]. 

Eyes were affected in 2 of our patients (18.8%). This rate was very different from 25 to 60% in other studies [12, 18, 20, 21]. In Chinese series ophthalmic involvement was more frequent in p-ANCA positive patients [23]. Skin lesion is uncommon symptom in GPA. In our case series the rate of skin involvement was 27.2%. In other study this rate was 11–48% [20–22]. 

## 5. Conclusion

GPA is a rare rheumatologic disorder in Iranian children with prevalence less than 1 per million children. The mortality rate was 18.1% in our study. GPA should be considered as a rare diagnosis in children with chronic respiratory involvement in concomitant with other organ involvement such as eye or renal involvement.

## Figures and Tables

**Table 1:** Clinical manifestations of patients with Wegener granulomatosis.

Clinical feature	Number	Percentage
Nonspecific complaints		
Malaise, fatigue	9	81.8
Fever	2	18.8
Weight loss	2	18.8
Lower respiratory tract (*n* = 7)		
Hemoptysis	2	18.1
Pleural effusion	1	9
Shortness of breath	2	18.1
Abnormal imaging	6	54.4
Upper respiratory tract (*n* = 9)		
Sinusitis	8	72.7
Otitis	3	27.2
Saddle nose	2	18.1
Renal (*n* = 4)		
Microscopic hematuria	4	36.3
Nephritic syndrome	1	9
Eyes	2	18.8
Skin	3	27.2
Gastrointestinal	3	27.2
Musculoskeletal		
Arthritis in knee and ankle	3	27.2
Nervous system		
Severe headache and seizure	1	9
Vasculitis	1	9
Venous thrombosis	1	9
